# Exploring Provider Perspectives on Gaps in Trauma-Informed Care Delivery Within the Acute Care Surgery Timeline at a Federally Qualified Health Center: Human-Centered Design Study

**DOI:** 10.2196/81004

**Published:** 2026-09-09

**Authors:** Angelica T McDaniel, Willow Frye, Neema Rashidi, Christiana D von Hippel, Marianna G Salvatori, Adrienne Greer, Manami Diaz Tsuzuki, Amanda Sammann

**Affiliations:** 1 UC Berkeley-UCSF Joint Medical Program, UC Berkeley, Berkeley, CA, United States; 2 The Better Lab, University of California, 2540 23rd St, 4th floor, San Francisco, CA, 94110, United States, 1 6282068000; 3 Department of Surgery, University of California, San Francisco, CA, United States

**Keywords:** trauma-informed care, perioperative medicine, human-centered design, burnout, provider experience

## Abstract

**Background:**

Trauma-informed care (TIC) education plays a pivotal role in preparing physicians to deliver care to patients who have experienced emotional and physical trauma, empowering patients to have agency in their care. The delivery of TIC by providers is especially important at Federally Qualified Health Centers (FQHCs), as their patients disproportionately experience trauma due to the impacts of socioeconomic determinants of health, including limited health care access, systemic racism, and housing insecurity. There is limited literature on how to adapt TIC practices in acute care settings, specifically for emergency medicine and surgical providers who deliver care in high-demand, time-constrained environments.

**Objective:**

The goal of this study was to understand the shared challenges faced by providers implementing TIC within acute care settings at an urban safety-net hospital.

**Methods:**

This exploratory qualitative study applied methods from the first stage of the human-centered design process, known as the inspiration phase, to identify the factors that influence providers’ decisions to provide or not to provide TIC within the acute care ecosystem. In this inspiration phase, we conducted 18 semistructured interviews with providers delivering surgical care at an FQHC in Northern California. Interviewers were purposefully sampled to represent the diversity of providers involved throughout the perioperative timeline, including physicians, nurses, social workers, psychologists, and de-escalation staff. Inductive thematic analysis of transcripts and subsequent insight statement generation were conducted to identify tensions between stakeholder needs and to elucidate design opportunities to improve their ability to deliver TIC.

**Results:**

Inductive analysis identified 21 unique themes and 7 insights that affect the effective implementation of TIC by providers in acute care settings. Time-constrained workflows, vicarious trauma, and provider burnout present significant barriers to its application. Furthermore, limitations in TIC training, hospital design, and security measures, translation challenges, and hierarchical structures all hinder its integration. Supportive measures include a strong desire to improve health outcomes for communities and patients at FQHCs.

**Conclusions:**

Through this first phase of the human-centered design methodology, we identified 7 key insights and design opportunities that lay the foundation for developing solutions to address gaps and enhance the delivery of TIC within safety-net hospitals.

## Introduction

Exposure to trauma within hospital populations is both widespread and complex, often stemming from chronic stressors that accumulate over time. According to the Substance Abuse and Mental Health Services Administration (SAMHSA), individual trauma “results from an event, series of events, or set of circumstances that is experienced by an individual as physically or emotionally harmful or threatening and that has lasting adverse effects on the individual’s functioning and physical, social, emotional, or spiritual well-being.” [1] Importantly, trauma is not limited to individual experience, as it can also be transmitted at the community and societal levels, including across generations. This is especially true for patients at Federally Qualified Health Centers (FQHCs), which are health care settings that receive federal funding to provide health care services to underserved populations, regardless of patients’ financial capacity. At these institutions, the patient population has an increased risk of trauma exposure due to socioeconomic challenges such as poverty, housing instability, and limited access to education [2]. One study found that approximately 80% of patients in safety-net hospitals have experienced 2 or more traumatic events, with 33% experiencing 5 or more [3]. Across trauma centers nationwide, the Improving Social Determinants to Attenuate Violence Workgroup within the American College of Surgeons has been actively addressing the root causes and impacts of trauma by promoting trauma-informed care (TIC) practices and curricular changes [4].

TIC is a strengths-based framework that is grounded in an understanding of trauma and the response to its impact, emphasizing the physical, psychological, and emotional safety of survivors to rebuild a sense of control and empowerment. According to the SAMHSA, TIC is a framework that health care systems can use to identify histories of traumatic experiences among patients and staff and enhance workflows by focusing efforts on developing policies and practices that prevent retraumatization [1]. In primary care settings, TIC has been used to improve provider and staff training, trauma screening protocols, trauma-informed interventions and resources, and staff burnout prevention [5]. Studies have shown that these practices allow providers to create a more comprehensive understanding of their patients’ histories, with visit lengths increasing by 5 minutes or less, 91% of the time [6]. However, in emergent settings (such as perioperative care, the emergency department, and the intensive care unit), time and workflow constraints pose additional obstacles to implementing TIC [7].

Surgical procedures are highly vulnerable experiences for patients, particularly regarding time-sensitive decision-making and bodily autonomy. The indications for surgical interventions, as well as the interventions themselves, carry the risk of causing psychological trauma in the preoperative, operative, and recovery periods. These environments place patients with unfamiliar providers, allot a limited time for decision-making, and compel patients to yield control to the health care system [8]. Frameworks and tools to understand and respond to this are critical for mitigating negative impacts and increasing opportunities for building patient-provider trust, facilitating patient-centered decision-making, and improving follow-up postoperatively with better outcomes. Although there has been an increase in research on TIC regarding patients, there has been little exploration into the insights of providers who deliver this care.

The research aim was to understand how to better adapt and implement TIC practices, education, and interventions in emergency care settings, particularly in FQHC surgical units, using interdisciplinary collaboration and design. While traditional qualitative research typically focuses on observing and understanding an environment, human-centered design (HCD) methodology was chosen to actively explore the perspectives and challenges of implementing TIC with the intent to create practical interventions [9]. HCD places users at the center of the design space, working toward co-designing solutions that incorporate the voices of stakeholders [10]. This methodology involves 3 phases: inspiration, ideation, and implementation. Each phase offers a stepwise approach to identifying the needs of the users, generating meaningful solutions, and turning these ideas into applicable outcomes. HCD has been used to develop prototypes in communities facing systemic barriers due to the prioritization of equal partnerships between stakeholders and researchers [11]. It has been used to improve documentation of geriatric care, reduce perinatal inequities, and design interventions for youth with HIV [10,12,13]. Incorporating users into the research team increases the likelihood of use, enhances accessibility, and improves the feasibility of the developed prototypes [14]. This paper focuses on the first inspiration phase, where an iterative process consolidates information gathered from interviews, ultimately laying the foundation for the development of interventions in subsequent phases.

## Methods

### Ethical Considerations

This study was approved by the University of California, San Francisco Institutional Review Board (IRB #22‐36409). The IRB approved a waiver of signed consent; participants were provided with an information sheet describing the study, and they verbally confirmed their agreement to participate over Zoom (Zoom Communications, Inc) prior to their interview. Participants were informed of their right to decline participation or withdraw at any time. Interview recordings were deidentified to protect participant privacy and confidentiality, and no identifying information is included in this manuscript.

### Study Setting and Participant Selection

This is an exploratory qualitative research study conducted at an FQHC in Northern California between 2022 and 2023 to understand clinicians’ perspectives on TIC within the acute surgical care setting.

Purposeful sampling [15] was used to recruit 18 stakeholders who represent the spectrum of providers with varying levels of experience and roles who work within emergency surgical care, from arrival at the emergency department to discharge. These included physicians and surgeons who direct clinical decision-making, nurses who provide continuous bedside care, social workers who address psychosocial needs, psychologists who support patients experiencing acute distress, and de-escalation staff who manage crisis situations. This diversity of roles was intentional in order to capture the fundamentally team-based nature of TIC. Individuals were recruited through provider and staff referrals at an FQHC emergency department and medical-surgical inpatient floors. No formal exclusion criteria were applied beyond failure to meet the inclusion criteria; all individuals who were referred and contacted by the research team agreed to participate and provided informed consent prior to their interviews. Interviews lasted approximately an hour and were conducted virtually through Zoom to accommodate clinical schedules and COVID-19 precautions in place at the time. Interviews were audio-recorded, deidentified for participant confidentiality, and transcribed for analysis.

### Qualitative Data Collection

Consistent with the HCD Inspiration phase, semistructured interviews were conducted using open-ended questions to elicit narratives identifying individual and systems-level challenges to providing TIC [16]. Interview guides were developed based on existing TIC literature and the SAMHSA framework [1,5], covering topics such as staff training, screening protocols, TIC interventions, and TIC implementation. These topics were used to understand our subjects’ perspectives on how TIC manifests in their daily work and the challenges they have encountered in its implementation. While all participants were asked the same core questions regarding their understanding of TIC, how they apply it in their daily work, and the barriers they encounter, role-specific probing questions were included to elicit contextually relevant examples.

### Qualitative Data Analysis

**Figure 1.**
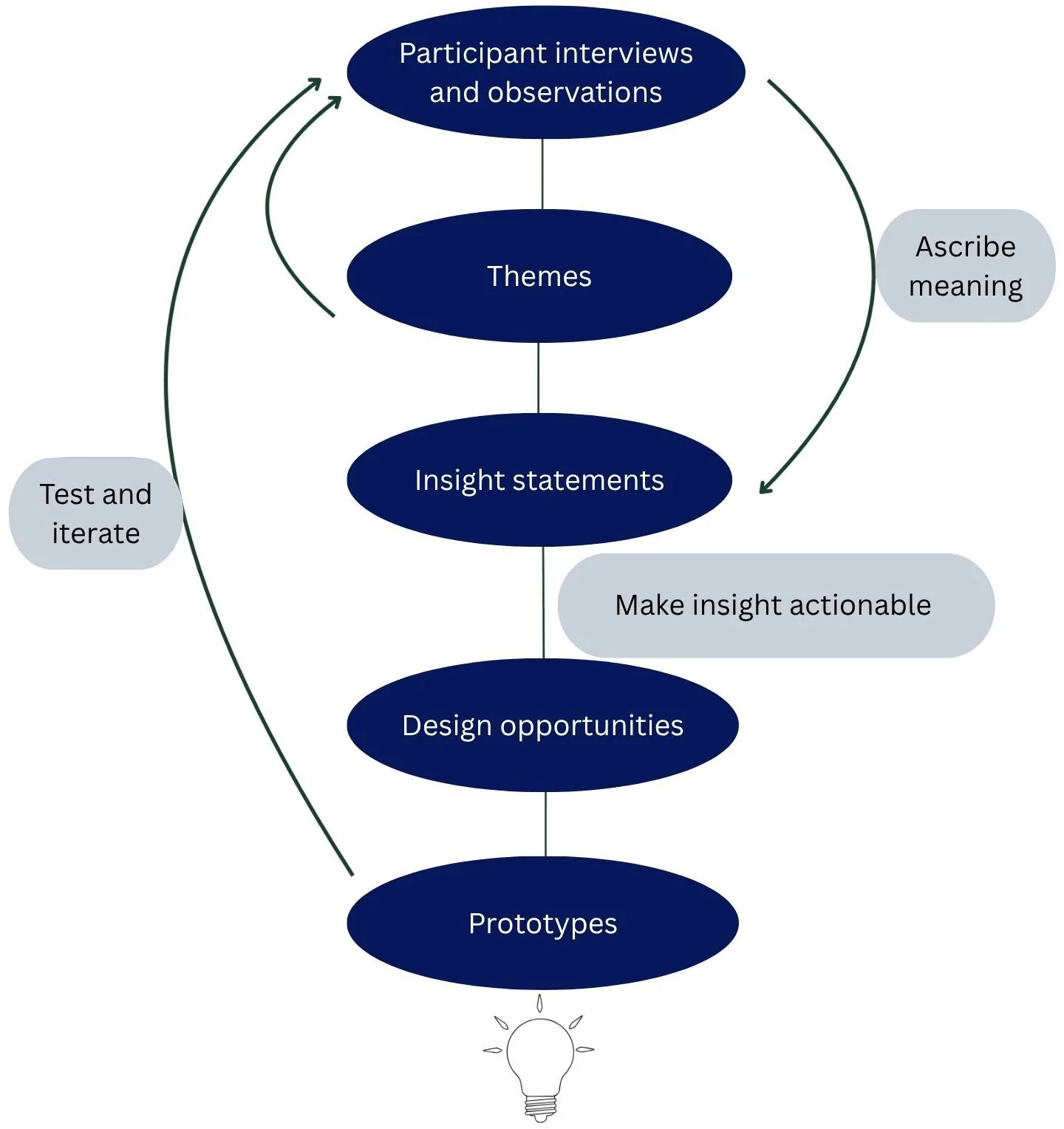
Human-centered design qualitative data analysis process diagram (previously published in the study by Lee et al [20], 2025).

A general inductive approach to thematic analysis [17] was conducted to identify key themes and exemplar quotes from deidentified interview transcript data. Two interview transcripts were selected by the interviewers for their representation of the sample’s diversity and the range of themes identified across all interviews. These transcripts were independently coded by 2 researchers (coder initials: AM and NR) to develop the initial codebook. To establish intercoder reliability, the researchers engaged in regular discussions after coding the representative interviews to ensure agreement on theme definitions and coding decisions. Once the initial codebook was defined, these researchers independently coded the transcripts using Dedoose (SocioCultural Research Consultants, LLC), a qualitative data analysis software, which facilitated the organization and analysis of the data [18]. Consistent with an inductive thematic analysis approach, the final codebook (Multimedia Appendix 1) was continuously refined throughout the analysis. Any new codes that needed to be added or existing codes that required redefinition were revised collaboratively by the team and applied consistently across all interviews. After interviewing 18 individuals, thematic saturation was achieved, and recruitment ceased. Thematic saturation was indicated by a lack of emergence of novel themes in new interview data; additionally, the number of interviews exceeded the commonly cited new information threshold of 10‐12 participants [19]. Themes, defined as inductively identified patterns of meaning derived from the interview data, were reassessed across the full set of interviews. Representative quotes were identified for each theme as empirical evidence of the observed patterns. Together, the themes and supporting quotes provided the foundation for insight statement development. Themes sharing common patterns or stakeholder concerns were grouped and synthesized into insight statements that captured the underlying tensions between stakeholder needs and systemic constraints. Each draft insight statement was written in actionable, user-centered language and iteratively reviewed and refined against supporting themes and quotes to ensure it remained grounded in the data. These insight statements define the user’s core motivations and needs, providing directed guidance for creating design opportunities (Figure 1). The design opportunities serve as starting points for potential interventions to address the issues identified under the insight statements. These inspiration phase activities are illustrated in Figure 1. Prototype development and iterative refinement, which comprise the subsequent ideation phase, will be addressed in future work.

## Results

### Qualitative Data Analysis

Eighteen interviews were conducted with a heterogeneous sample of individuals who work across the spectrum of emergency surgery, including physicians, nurses, social workers, psychologists, and de-escalation staff (Table 1). Thematic analysis of these interviews revealed 21 themes, organized under 7 overarching insight statements. These are presented alongside supporting quotes and design opportunities that highlight the obstacles and tensions involved in delivering TIC (Textbox 1).

**Table 1.** Participant demographics (N=18).

Participant role	Count, n (%)	Female, n (%)	0‐5 years experience, n (%)	5+ years experience, n (%)
Total	18 (100)	15 (83.3)	1 (5.6)	17 (94.4)
Physicians (total)	9 (50)	8 (88.9)	1 (11.1)	8 (88.9)
Residents	1 (5.6)	1 (100)	1 (100)	0 (0)
Fellows	2 (11.1)	2 (100)	0 (0)	2 (100)
Attendings	6 (33.3)	5 (83.3)	0 (0)	6 (100)
Nurses (total)	6 (33)	4 (66.7)	0 (0)	6 (100)
Nurse practitioners	2 (11.1)	2 (100)	0 (0)	2 (100)
Registered nurses	4 (22.2)	2 (50)	0 (0)	4 (100)
Social workers	1 (5.6)	1 (100)	0 (0)	1 (100)
Psychologists	1 (5.6)	1 (100)	0 (0)	1 (100)
De-escalation staff	1 (5.6)	1 (100)	0 (0)	1 (100)

Textbox 1. **Themes, quotes, insights, and design opportunities.**

**Theme 1a: Implementation of trauma-informed care (TIC) takes time**

- **Quote**: “One of the biggest and sometimes hardest things to do in surgery is to spend time. The time that you need with patients to understand what’s going on and counsel, and educate, and explain to them their options appropriately. I think that is really hard, especially as a resident.”—General Surgery Resident Physician 1

**Theme 1b: The clinical workflow does not support the time required for TIC**

- **Quote**: “People don’t want to stop and slow down and be thoughtful because that takes more time, and the system doesn’t really reinforce taking time. The system reinforces productivity.”—Trauma Recovery Center Staff 1

**Theme 1c: Health care has a productivity-driven focus over patient-centered care**

- **Quote:** “The goal in our health care system is not to provide care. It is to make money. So, our health care system in the United States is in direct conflict with itself. It makes more money the less care it provides.”—Emergency Medicine Nurse 1

**Insight 1:** TIC requires excess time for provider education and clinical implementation, which the productivity-driven hospital workflow does not accommodate.

**Design opportunity:** integrate TIC training within hospital workflows and medical education to support productivity and respect time constraints.

**Theme 2a: Providers are burned-out without support structures**

- **Quote:** “There is some forced compartmentalization here because I need to go take care of the next person in front of me...Yes, this horrible thing happened. I just did all of this very invasive, emergent, intense stuff. And I’m just going to have, just put it in a box and think about it, process, talk about it later because it can’t happen right now. Because there’s somebody else in front of me I need to take care of who needs 100% of my attention.”—Acute Care Surgery Fellow Physician 1

**Theme 2b: Burned-out providers struggle to implement TIC**

- **Quote:** “I feel like I’m fighting all the time. I’m fighting for resources for my patients. I’m fighting for resources for the staff. I am fighting for vulnerable populations. I feel like I’m fighting all the time. And it’s wearing me out.”—Emergency Medicine Nurse 2

**Theme 2c: Providers believe patients are negatively impacted by the lack of TIC**

- **Quote:** “The patient told me, ‘Well, nobody ever in the health care system ever smile at me.’ And they told me, ‘You’re the first one smiling at me.’ And I think to them it was a way to say, ‘Well I never felt that there was any compassion for me in the health care system.’ And to some extent they felt that they were being perceived as some sort of a burden.”—Anesthesia Attending Physician 1

**Insight 2:** Providers who do not receive support to address their own vicarious trauma and burnout do not have the personal resources to prioritize TIC.

**Design opportunity:** Create supportive structures for providers to process vicarious trauma and mitigate burnout, enabling them to sustain and prioritize TIC.

**Theme 3a: TIC training lacks a consistent, core curriculum**

- **Quote:** “Everybody should have it the same way. And I know people don’t like that. They like having the freedom, but I think if the idea is to start out with this standard and then have good clinical evidence and data while you deferred from it, that’s okay. I’m not saying that everybody should do the same thing, but I think that we should start out with some of the best. And if we’re going to do less than that or more than that, we need to have data as to why.”—Interventional Radiology Nurse 1

**Theme 3b: TIC training lacks specialty-specific considerations**

- **Quote:** “I was just doing a trauma-informed course. It was a training the trainer type of course that the ACS is starting to roll out. And this idea that you can ask open-ended questions or ask people things like, ‘What can we do for you?’ Or we can approach somebody in a trauma-informed way, sitting down to talk to them without it taking more time. I think that’s probably unrealistic to say. To say that it’s not going to take more time than what that takes, is just at face value not believable.”—Acute Care Surgery Fellow 2

**Theme 3c: Traditional medical training does not always incorporate TIC**

- **Quote:** “TIC is definitely not something that you are taught in any of your training. I think that’s also part of the problem. Because when you’re in nursing school, you get assigned to all of the quote unquote ‘cooperative or nice patients.’ You don’t get to experience patients who may not necessarily go along with the care plan. Then when you enter the workforce, you’re shocked that, oh, you don’t want to take your medicines?”—Medical-Surgical Nurse 1

**Insight 3:** TIC training is too inconsistent to be widely adopted and too generic to address specialty-specific needs, making it difficult for clinicians to apply in real-world care.

**Design opportunity:** Design a flexible TIC training model that combines a standardized core curriculum with specialty-specific modules, enabling both broad adoption and practical implementation across diverse clinical settings.

**Theme 4a: Providers believe hospital design can negatively impact patients’ well-being**

- **Quote: “**Just imagine what some individuals who have experienced being in prison, when they’re walking through the doors of (the hospital) and the first people they encounter are police officers, and then they have to call in and they have to go through multiple different locked doors to get to their family member. It feels very much the same.”—Anesthesia Attending Physician 1

**Theme 4b: Providers believe hospital safety measures can negatively impact patients’ well-being**

- **Quote:** “I’m not saying that there should not be any police. I think in some instances you may need the police because patients come with knives and guns and things of that nature. But I really do feel like we need to invest in other forms of care to help deescalate patients so we don’t get there, so we don’t need the police there. But it can definitely be triggering for patients. Because you don’t know what someone’s history is, or how they have interacted with the police. If this is supposed to be a place of healing, having them here doesn’t necessarily align with it.”—Medical-Surgical Nurse 3

**Theme 4c: Compliance with hospital protocol is not in concordance with being trauma-informed**

- **Quote:** “Then the doctor says, ‘We’re going to restrain her.’ ‘Wait a second, what are you talking about? We’re not going to restrain her. She was a victim of sex trafficking. Held hostage. Recently incarcerated. Why are we going to restrain her?’ The situation was bananas. I just could not believe what was happening.”—Medical-Surgical Nurse 1

**Insight 4:** Hospital architecture, security measures, and compliance are meant to ensure safety, but instead make patients with trauma feel unsafe, undermining the goals of TIC.

**Design opportunity:** Create physical hospital environments that balance structural requirements, security, and compliance with trauma-informed design principles to ensure a psychological sense of safety for patients.

**Theme 5a: Using translation services impedes human connection between patient and provider**

- **Quote:** “I think language barriers are huge. And I think people are doing a good job of using translators and all that, but I even sometimes feel like I’ll sit down for a long time with a translator and I still can’t have a conversation where I feel like we’re understanding each other, like the patient and I are understanding each other through a translator. I feel like that can be a really big barrier sometimes to having those meaningful conversations with patients.”—General Surgery Resident Physician 1

**Theme 5b: Direct language translation is not always inclusive of cultural translation**

- **Quote:** “One of the medical student interns who spoke fluent Spanish, she wasn’t certified as a bilingual interpreter, but we were consenting a patient for a cholecystectomy. And she was listening to the phone interpreter we were using to do the actual legal informed consent and everything. And the interpreter said something and she just shook her head and was like, ‘No, no. This is actually what we’re saying.’”—Acute Care Surgery Fellow 1

**Theme 5c: Providers are concerned about providing suboptimal care due to communication barriers**

- **Quote:** “But just having to use phone interpreters, that I think a lot of things are lost in interpretation. And there are definitely, I can think of a handful of times where I felt like I was having, say a pretty thorough preoperative discussion just for something entirely elective, like a cholecystectomy, and basically having the patient come back to me at the end of a 20 minute conversation, when I’ve clearly said through the interpreter, ‘We’re going to be taking out your gallbladder.’ And the patient says, ‘So what exactly are you going to be doing in surgery?.’.. I know this inherently is going to take longer than, say if I’m just having a conversation with somebody, who their first language is also English. But also making sure that I have pretty much that same conversation. That I’m not cutting corners or leaving out details because I’m having to go through an interpreter.”—Acute Care Surgery Fellow 2

**Insight 5:** When translation is inconsistent or lacks cultural nuance, providers struggle to build trust and worry they are delivering lower-quality care to non–English-speaking patients.

**Design opportunity:** Enhance the quality and consistency of translation services to help providers deliver clear, compassionate, and patient-centered care to non–English-speaking patients.

**Theme 6a: Trust is achieved by giving patients a voice**

- **Quote:** “Imagine being a patient, not feeling like yourself and away from all your people, away from all your normal ways of coping with things. Your modesty ripped away from you because you’re in this silly gown and people keep coming in and the whole waking up thing, being woken up at four in the morning by a door creaking open, all of it you have to realize is not a good experience. It’s all traumatic. Even if you’re here for a lap coley. It’s all just uncomfortable. So I think first and foremost, just actually acknowledging that like, ‘I hear you, this sucks. It’s not fun to be here.’ And going through those things. ‘I know you don’t like getting up in the middle of the night.’ Even having that question, ‘What would make this easier for you? There’s things that have to happen. There’s things that maybe we can work around. What would make this easier for you?’ And you can be even honest.”—Surgery Nurse Practitioner 1

**Theme 6b: Hospital hierarchies hinder the ability to incorporate the voices of staff working closest to patients**

- **Quote:** “Because I really do try to advocate for patients. Some nurses, they’re just scared, I think. Because they’re just like, ‘Well, the doctor said. The doctor said.’ It’s like, okay. But it’s okay to ask questions. It’s okay to challenge the doctor. You’re doing it in a very professional manner. But you’re looking out for the best interest of your patient. But the problem is, if everyone just goes along with the plan and nobody questions things, it doesn’t always work out for the patient.”—Medical-Surgical Nurse 2

**Theme 6c: Hospital hierarchies hinder the ability to incorporate the voices of patients**

- **Quote:** “A lot of our patients I feel like just want more control over their care. If you sit down and you talk to patients about what’s going on and how they feel it might be best to manage it, I feel like you can get their buy-in a little bit more. Versus talking at them. Encouraging them to participate in their care. These are adults and they can make decisions. I just feel like sometimes we don’t allow that. Our system doesn’t allow for that. It’s very rigid.”—Medical-Surgical Nurse 1

**Insight 6:** Providers earn patient trust through involving them in their care, but medicine’s hierarchical structures can impede this trauma-informed approach.

**Design opportunity:** Integrate diverse team viewpoints to address patient concerns and foster improved communication and stronger relationships.

**Theme 7a: Providers work in Federally Qualified Health Centers (FQHCs) to care for under-served and at-risk patients**

- **Quote:** “And I was drawn to this hospital because of the way the patient population was treated there, I’m not someone who believes that you should be treated differently based on the thickness of your wallet when it comes to health care.”—Emergency Medicine Nurse 1

**Theme 7b: Providers persevere through the hardship for the benefit of their community**

- **Quote:** “For whatever reason I’ve experienced PTSD, burnout, all kinds of things and I have worked very hard to take care of myself to continue. Cause the mission of this hospital, the population that we take care of, is a very needy population. In need of support, in need of family, and in need of a lot of compassion. So I continue to feel like I’m relevant, even though sometimes I look in the mirror and I say I don’t know if I’m still relevant.”—Emergency Medicine Nurse 2

**Theme 7c: The toll of working for FQHCs pushes providers to leave**

- **Quote:** “I’m definitely considering leaving. The emotional toll that my shifts take on me now are significant. I’ve made efforts to affect change by becoming more active in the union, which just means that I’ve made myself apparently an enemy of the administration and it’s made things harder.”—Emergency Medicine Nurse 1

**Insight 7:** The more deeply providers care about their patients and mission, the more likely they are to struggle to sustain their sense of purpose over time in high-demand safety-net settings.

**Design opportunity:** Create systems and support structures that help providers at safety-net hospitals sustain their sense of mission and commitment to public health, despite the demands of patient care.

### Insight 1: TIC Requires Additional Time for Provider Education and Clinical Implementation, Which the Productivity-Driven Hospital Workflow Does Not Accommodate

A total of 10 (56%) interview participants stated that an assembly-line structure of hospital workflows does not adapt effectively to the diverse trauma-informed needs of providers and patients. Providers experience a persistent tension between their desire to deliver holistic, person-centered care and the time constraints imposed by a productivity-driven environment. This tension limits opportunities to develop personalized care plans, build meaningful provider-patient relationships, and engage patients in the deeper conversations necessary to uncover past experiences, apprehensions, and emotional needs prior to care. When a fast-paced, profit-driven hospital structure systematically prioritizes efficiency over emotional engagement, the psychological and relational dimensions of patient care are consistently overlooked.

Design opportunity 1: Integrate TIC training into hospital workflows and medical education to support productivity while respecting time constraints.

Interventions to improve TIC within a production-oriented hospital work environment should focus on uplifting patient experiences without coming at the expense of providers’ time and mental capacity. These could include integrating training into previously scheduled didactic time or providing support systems that alleviate the burden on providers, reducing the need for them to manage TIC independently.

### Insight 2: Providers Who Do Not Receive Support to Address Their Own Vicarious Trauma and Burnout Do Not Have the Personal Resources to Prioritize TIC

Providers working in high-trauma environments face a continuous cycle of emotional suppression that erodes their well-being and long-term commitment to the field. A prominent pattern across interviews was that acute care requires providers to set aside their own emotional responses to deliver consistent care to each patient, yet they often lack adequate opportunities for recovery or resolution. For providers working primarily in acute care settings, where patient encounters are concentrated in moments of crisis, limited visibility into patient recovery and positive outcomes can deepen this emotional toll and distort their broader sense of purpose in their work. When burnout takes hold, providers lose the capacity for empathy, compassion, and sustained attention that TIC demands. The consequences extend beyond individual providers: when providers lack the personal resources to sustain trauma-informed practices, patients feel the absence of compassion in their care, perceiving themselves as unseen or a burden within the health care system. Together, these conditions make it increasingly difficult for providers to sustain the personal and professional resources necessary to prioritize TIC.

Design opportunity 2: Create supportive structures for providers to process vicarious trauma and mitigate burnout, enabling them to sustain and prioritize TIC.

Interventions that provide both the time and resources necessary to reflect on challenging experiences in the hospital setting are crucial. Providing structured opportunities for debriefing, peer support, or access to mental health resources can help mitigate the stress and burnout associated with these experiences. These support mechanisms offer providers the opportunity to improve personal resilience and to engage in their careers with greater compassion and empathy.

### Insight 3: TIC Training Is Too Inconsistent to Be Widely Adopted and Too Generic to Address Specialty-Specific Needs, Making It Difficult for Clinicians to Apply in Real-World Care

Interviews revealed both an appreciation for TIC and notable gaps in the current structure and delivery of training. Providers described the need for a standardized foundational curriculum that establishes clear expectations and a shared framework, with the understanding that evidence-based deviations from that standard are appropriate when supported by data. However, foundational knowledge alone was not considered sufficient in practice. One provider who had participated in formal TIC training found that certain recommended approaches, such as asking open-ended questions or engaging patients in a trauma-informed manner, felt unrealistic within the time constraints of their specialty. This highlighted a broader need for training that accounts for the specific demands of different clinical settings while allowing providers the flexibility to adapt their approach to each unique patient situation. Further complicating adoption, TIC is not consistently incorporated into formal health care education. Seven (39%) interviewees described their trauma-informed practices as largely intuitive or drawn from personal and patient experiences rather than structured instruction, reflecting a broader gap between what training prepares them for and what they encounter in clinical practice.

Design opportunity 3: Design a flexible TIC training model that combines a standardized core curriculum with specialty-specific modules, enabling both broad adoption and practical implementation across diverse clinical settings.

Improvements to TIC training should focus on teaching and requiring core components that allow for personalized education, making it easier to meet the differing demands of each specialty and provider role. This could include adding unique modules and courses for learners at all levels of training and across fields.

### Insight 4: Hospital Architecture, Security Measures, and Compliance Are Meant to Ensure Safety, but Instead Make Patients Who Have Experienced Trauma Feel Unsafe, Undermining the Goals of TIC

Providers described a fundamental tension between hospital safety requirements and the goal of making patients feel safe. One interviewee described how the physical layout of hospitals, from security checkpoints to locked entry points, and a uniformed police presence, can mirror environments associated with incarceration, creating a sense of threat for patients before care even begins. While providers recognized that law enforcement presence is sometimes warranted given safety risks, they also acknowledged that it introduces an inherent contradiction in a space meant for healing, particularly for patients whose histories include negative interactions with law enforcement. Rather than defaulting to security personnel, providers called for greater investment in de-escalation approaches that address the root of patient distress. This tension extended into clinical decision-making as well. Providers described situations in which standard hospital protocols were applied without consideration of a patient’s trauma history, such as the use of restraints on a patient with a documented history of sex trafficking or incarceration. Together, these dynamics place providers in the difficult position of navigating between institutional compliance and their commitment to TIC.

Design opportunity 4: Create physical hospital environments that balance structural requirements, security, and compliance with trauma-informed design principles to ensure a psychological sense of safety for patients.

A third of participants remarked in their interviews that to prevent retraumatizing patients, it is essential to have surroundings where both providers and patients feel safe, highlighting how TIC can be built into the design, structure, and regulations of the environment as well.

### Insight 5: When Translation Is Inconsistent or Lacks Cultural Nuance, Providers Struggle to Build Trust and Worry They Are Delivering Lower-Quality Care to Non–English-Speaking Patients

Language and cultural barriers emerged as a source of stress for providers, complicating their ability to build trust and ensure that patients fully understood their care. Providers noted that even when translation services were used consistently, they did not always result in genuine mutual understanding between patients and providers. Interviewees highlighted that cultural understanding is not always guaranteed when using these services and that, without sufficient time, it becomes more challenging to build trust and communicate clearly. Due to the heightened time constraints associated with using translators, many providers shared their discontent with the quality of their appointments, expressing that they felt they were providing suboptimal care to patients who speak languages other than the providers’ own.

Design opportunity 5: Enhance the quality and consistency of translation services to help providers deliver clear, compassionate, and patient-centered care to non–English-speaking patients.

Overall, providers emphasized in their interviews the difficulty and importance of holding nuanced care conversations with non–English-speaking patients to account for the emotional, psychological, and cultural elements that are essential to build the trust required for TIC. Interventions should be designed to address these communication gaps between providers, translators, and patients to foster stronger relationships and improve collaboration.

### Insight 6: Providers Earn Patient Trust Through Involving Them in Their Care, but Medicine’s Hierarchical Structures Can Impede This Trauma-Informed Approach

The hospital experience can be disorienting for patients, who often find themselves distanced from their sense of control, comfort, and familiar coping mechanisms. Providers described how simply acknowledging this discomfort can be a foundational step in building trust and creating space for collaboration. Providers noted that when patients are treated as active participants in their own care rather than passive recipients of it, they are more likely to engage with and follow through on their care plans. However, structural barriers consistently undermined this approach. One provider described, for example, a culture of deference within hospital hierarchies that discouraged staff from questioning decisions even when doing so was in the patient’s best interest. Another interviewee described a system too rigid to accommodate meaningful patient input, one that defaulted to talking at patients rather than with them, and limited the kind of shared decision-making that TIC requires. These examples point to a broader disconnect between what providers recognize as effective, trust-building practice and what the current structure of hospital care allows them to do.

Design opportunity 6: Integrate diverse team viewpoints to address patient concerns and foster improved communication and stronger relationships.

Relationships between patients and provider care teams require interventions that support ongoing communication and provide avenues for voicing opinions and concerns. Since TIC relies on a team-based approach, fostering these relationships will enhance its efficiency and implementation.

### Insight 7: The More Deeply Providers Care About Their Patients and Mission, the More Likely They Are to Struggle to Sustain Their Sense of Purpose Over Time in High-Demand Safety-Net Settings

Ten (56%) providers described choosing to work at this safety-net hospital as a deliberate values-driven decision, motivated by a belief that quality health care should be accessible to everyone regardless of their financial or social circumstances. For some, this commitment was deeply personal, rooted in their own experiences of loss, inadequate support, or systemic failure, and shaped by a determination to ensure their patients did not experience the same. Yet sustaining that commitment has come at a significant cost. The emotional weight of the work, compounded by institutional barriers that discourage advocacy and resist change, has prompted some providers to consider leaving. One provider described how attempting to address these issues from within had at times led to conflict with hospital administration, making an already difficult environment even harder to navigate. Together, these experiences reflect a deeper tension between the passion that draws providers to this work and the conditions of a system that too often fails to sustain them within it.

Design opportunity 7: Create systems and support structures that help providers at safety-net hospitals sustain their sense of mission and commitment to public health, despite the demands of patient care.

Interventions that help sustain the drive and morale of safety-net hospital workers include methods that support providers in furthering their interests in the field of public health and offer recognition for the work they do.

## Discussion

This study demonstrates that the successful implementation of TIC in acute care settings is not limited by provider willingness, but by structural conditions that constrain their ability to incorporate TIC into clinical care. Time-pressured workflows, insufficient staffing, productivity-driven priorities, and limited access to tailored training collectively create an environment where TIC is difficult to operationalize, even when providers recognize its importance. These constraints contribute to and are exacerbated by burnout and unaddressed moral distress, further reducing providers’ capacity to consistently deliver TIC. In response, many clinicians rely on personal intuition and prior experiences rather than formal training, highlighting a gap between institutional preparation and real-world demands. Additionally, features of the care environment such as rigid protocols and security presence may inadvertently undermine patients’ sense of safety, directly subverting the goals of TIC. Despite these barriers, providers consistently express a strong commitment to their patients and communities, suggesting that effective solutions must align system design with this intrinsic motivation to enable sustainable, trauma-informed practice.

These findings highlight a self-reinforcing cycle in which exposure to patient trauma and moral distress diminishes providers’ capacity to deliver TIC, thereby contributing to further patient trauma and adversely affecting patient outcomes. Repeated exposure to patient trauma can lead to secondary traumatic stress among providers, reducing their ability to consistently deliver compassionate, TIC [21]. The tension between providers’ strong desire to deliver TIC and the real-world constraints that prevent them from practicing in alignment with their values results in further moral injury, which is a critical and underrecognized barrier to TIC [22]. The resulting threats to workforce wellbeing constitute a patient safety concern, as they are associated with decreased quality of care, increased medical errors, and reduced patient satisfaction [23]. Collectively, these findings underscore that workforce wellbeing and TIC are essential and interdependent, serving as foundational prerequisites for the delivery of safe, equitable, and TIC.

These findings also demonstrate how the unique context of high-acuity, team-based emergency surgery amplifies known barriers to TIC implementation. The need for rapid decision-making and tightly coordinated multidisciplinary care intensifies the tension between efficiency and human-centered care, making consistent application of TIC particularly challenging [24]. The trifecta of burnout, inadequate support, and structural barriers undermines the reliable integration of TIC principles in high-stakes surgical care, at a time when patients are especially vulnerable to trauma.

Despite these challenges, the foundational goals of TIC, originally developed to recognize and respond to trauma, remain highly relevant given the pervasive prevalence of trauma [1]. A trauma-informed approach is particularly critical in FQHC settings, where patients are disproportionately affected by prior traumatic experiences [3]. Although neither safety-net hospitals nor TIC alone can resolve structural inequities in health care, both are grounded in a shared commitment to advancing health equity and ensuring access to compassionate, patient-centered care.

While our data provide valuable insights, it is important to acknowledge limitations in our study design. The scope of this study only included participants from one FQHC in California. Our study population was recruited primarily through participant referrals, which limited our ability to obtain a sample that reflects the full diversity of the hospital’s occupational demographics. This may reflect self-selection bias inherent to voluntary recruitment methods, as individuals with a preexisting interest in TIC may have been more likely to participate. Additionally, our sample included a substantial proportion of attending physicians. While trainees and other providers are often more directly involved in the initial management of trauma patients, attending physicians play a key role in shaping departmental culture and the adoption of trauma-informed practices. Future studies should incorporate more trainees in their study populations to better understand frontline implementation. Our data may be transferable but not generalizable to other hospital systems, as health care providers’ experiences may vary across different geographical locations and workplace environments.

Future work will also focus on understanding the perspectives of patients receiving treatment from acute care surgery teams, gathering data on their perceptions of how TIC is delivered in the hospital setting, and identifying which support systems enhance this type of care. Using data from both provider and patient perspectives will enable a more holistic understanding that will allow future researchers to develop prototypes to further improve TIC implementation in FQHCs. By understanding this context, we can gain valuable insights into how to improve the effectiveness and accessibility of TIC, thereby creating the steps necessary to begin the next stage of HCD, the ideation phase. In this phase, prototypes will be developed to address these issues, ultimately improving patient outcomes and fostering a more supportive health care environment.

## Supplementary material

10.2196/81004 Multimedia Appendix 1 Interview codebook.

## Abbreviations

FQHC: Federally Qualified Health Center
HCD: human-centered design
SAMHSA: Substance Abuse and Mental Health Services Administration
TIC: trauma-informed care
