# Supplementary material for: Exploring Provider Perspectives on Gaps in Trauma-Informed Care Delivery Within the Acute Care Surgery Timeline at a Federally Qualified Health Center: Human-Centered Design Study
Source: JMIR Perioper Med. 2026 Sep 9;9:e81004. doi: 10.2196/81004 (PMC13557612; doi:10.2196/81004)
Supplement: Supplementary file 1 [file periop-v9-e81004-s001.docx]

**Supplementary Material: Interview Codebook**

| **Parent Code** | **Child Code** | **Description of Code** |
| --- | --- | --- |
| **Hierarchy** |  | Moments when hierarchy in hospitals are mentioned |
| **Historical Trauma** |  | When previous traumas of the patient or provider affect care |
| **Provider Experience** |  | Moments when providers share about their experience providing care |
|  | Provider Well-being Techniques | When providers mention what techniques they use to maintain wellbeing |
|  | Provider Fear | Moments when providers acknowledge their fear interrupting care |
|  | Burnout | Moments when providers exemplify burnout within their current role |
|  | Interprovider relationship | Moments when providers describe their relationships with their colleagues |
| **De-escalation Techniques** |  | Instances when deescalation techniques are implemented in a reaction to an event |
| **Safety Net Hospital Characteristics** |  | Moments when the environment of a safety net hospital affect care |
| **Trauma Informed Care** |  | Moments that discuss care that providers label as trauma informed care |
|  | Current Trauma Informed Practices | Moments that exemplify how providers currently implement trauma informed care as preventative and reactionary measures |
|  | Current Training of Providers | Moments when providers describe how they learned trauma-informed care strategies, informally or formally |
|  | Trauma-Informed Care Description | Providers description of trauma informed care or techniques they think would improve trauma-informed care |
| **Motivations for Practice** |  | Moments when providers describe why they chose to work in their field and specialty |
| **Systemic Conditions** |  | Moments that the hospital system interferes with care |
|  | Limited Support | Moments when providers mention limited support to help them provide adequate care |
|  | Limited Time | Moments when providers describe time pressure during clinical encounters |
|  | Law Enforcement | Moments when law enforcement affected clinical situations |
|  | System Rigidity | Moments when the hospital system prevents how the provider would originally chose to provide care |
